# Artifact interactions retard technological improvement: An empirical study

**DOI:** 10.1371/journal.pone.0179596

**Published:** 2017-08-04

**Authors:** Subarna Basnet, Christopher L. Magee

**Affiliations:** 1 SUTD-MIT International Design Center, Singapore University Technology and Design, Singapore, Singapore; 2 SUTD-MIT International Design Center, Massachusetts Institute of Technology, Cambridge, Massachusetts, United States of America; 3 Department of Mechanical Engineering, Massachusetts Institute of Technology, Cambridge, Massachusetts, United States of America; 4 Institute for Data, Systems and Society, Massachusetts Institute of Technology, Cambridge, Massachusetts, United States of America; Tianjin University, CHINA

## Abstract

Empirical research has shown performance improvement of many different technological domains occurs exponentially but with widely varying improvement rates. What causes some technologies to improve faster than others do? Previous quantitative modeling research has identified artifact interactions, where a design change in one component influences others, as an important determinant of improvement rates. The models predict that improvement rate for a domain is proportional to the inverse of the domain’s interaction parameter. However, no empirical research has previously studied and tested the dependence of improvement rates on artifact interactions. A challenge to testing the dependence is that any method for measuring interactions has to be applicable to a wide variety of technologies. Here we propose a novel patent-based method that is both technology domain-agnostic and less costly than alternative methods. We use textual content from patent sets in 27 domains to find the influence of interactions on improvement rates. Qualitative analysis identified six specific keywords that signal artifact interactions. Patent sets from each domain were then examined to determine the total count of these 6 keywords in each domain, giving an estimate of artifact interactions in each domain. It is found that improvement rates are positively correlated with the inverse of the total count of keywords with Pearson correlation coefficient of +0.56 with a p-value of 0.002. The results agree with model predictions, and provide, for the first time, empirical evidence that artifact interactions have a retarding effect on improvement rates of technological domains.

## Introduction

**Fig 1:**
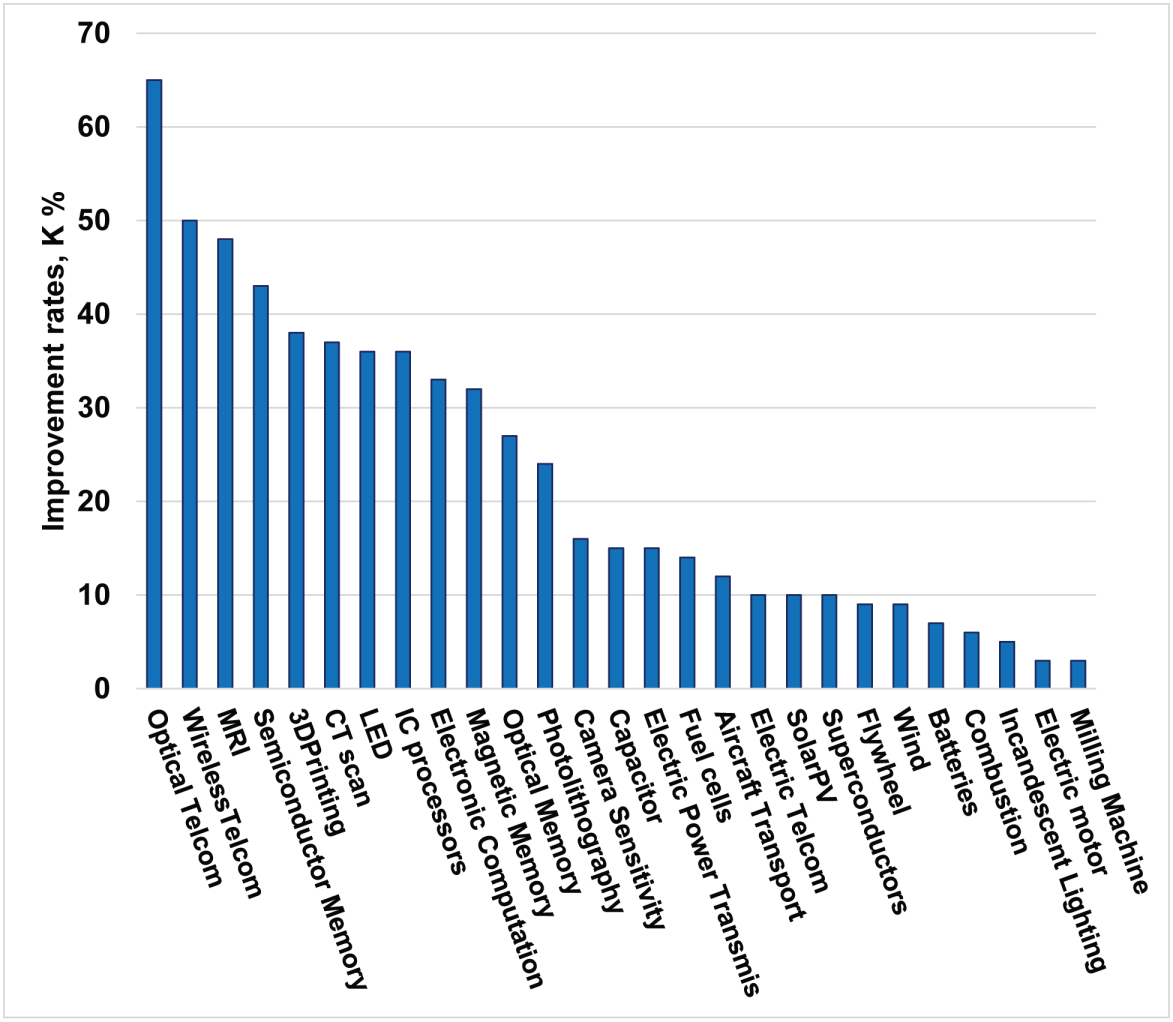
Annual performance improvement rates (K_J_) for 27 domains. The value of K_J_ for each domain is the slope of a linear curve fitted to log of the performance metric versus time using data from 1976 until 2013. The rates vary widely, from 3 to 65 percent. Adapted from Magee, Basnet, Funk and Benson 2016.

Within the large and complex field of technical change, empirical research has demonstrated technological performance improves exponentially over time, but with widely varying improvement rates across the domains. In addition, knowledge of how and at what rate performance of a given technology improves is important for corporate product planners and designers, policy makers, and investors [1–7]. To improve consistency and reduce ambiguity in measurement of technology performance and its improvement, we have chosen technological domains as the unit of analysis, which are defined as a set of designed artifacts that utilize a recognized body of knowledge to achieve a specific generic function [7]. The artifacts considered can be products, software, or processes. The body of knowledge is principally scientific and engineering knowledge of particular relevance to the domain of interest; so each functional category (e.g., energy storage) is decomposed into technological domains (e.g., electrochemical battery, capacitor, and flywheel) based on the scientific knowledge utilized by the artifacts considered. The performance metric of a technological domain, defined from the perspective of users of technology, is a composite indicator which includes essential functional outputs and a resource constraint (e.g., cost, volume or mass of the artifact) important to the users [7]. The performance metric is formulated so that as the functional outputs improve the performance metric increases, and is expressed per unit of resource considered. The available data has been, accordingly, adapted to construct performance data for 71 metrics in 27 domains [7]. The analysis of empirical data has demonstrated that performances of the 27 technological domains considered improve exponentially, but the annual improvement rates (K_J_) vary widely ranging from 3 to 65 percent (shown in Fig 1 for the most reliable estimates).

An artifact typically consists of number of components, where the components work together to achieve the overall function of the artifact. In attempting to improve performance of the artifact, components are repeatedly modified [8]. When a component, say B, is modified, it affects other components, such as C, D and E, requiring changes in them. If the overall performance of the artifact degrades after the changes, instead of improving, such changes are not acceptable, and additional iterations have to be initiated with components B, C, D, and E until an acceptable solution is found. Such an interaction requires negotiations going back and forth in order to improve performance by resolving dependency and conflicts. A special case arises when component B changes, but C, D and E are not affected or they can accommodate any degree of change in B. In such a case, there is no need for back and forth negotiation, in short, there is no interaction.

A quantitative model of the effect of such interactions on technological improvement was developed first for simple artifacts and changes in cost [8]. For a simplified artifact with interaction parameter *d* for each component (defined as the average number of components *affected* by a design change in a given component including itself, with effects as described in the prior paragraph), McNerney et al.’s treatment [8] for unit cost results in the following relationship:

$$\frac{dC}{dm}=-B\cdot C^{d+1}$$ (1)

Where, C = unit cost normalized with respect to initial cost, m = number of attempts, d = interaction parameter, B = constant. The normalized unit cost C is 1 or less by definition, so increases in d in Eq 1 results in less improvement per attempt.

Considering a broader framework, Basnet and Magee [9] and Basnet [10] have extended these results into the form tested here. Their overall result is summarized by an equation giving improvement rate of a technological domain based on the three factors on the right hand side of Eq 2:

$$K_{J}=\frac{d\text{ ln }Q_{J}}{dt}=\left(\pm \right)A_{J}\frac{1}{d_{J}}K$$ (2)

Where Q_J_ is the performance of a domain J and K_J_ its performance improvement rate (or, the slope of the performance curve in a semi-log plot with time). A_J_, and d_J_ are respectively a set of scaling and interaction parameters specific to a domain. K is domain independent, and represents the rate of growth in the number of individual operating ideas (IOI) generated through a combinatorial analogical transfer process encompassing all of science and technology. These IOI are then assimilated into the components of domain artifacts, where the influence of interaction and scaling parameters manifest. The equation predicts that K_J_ (improvement rate) is proportional to the inverse of the interaction parameter d_J_, stating that domains with a higher average number of interactions (d_J_) will be retarded leading to slower improvement rate. However, is there evidence that demonstrates artifact interactions retard improvement rates of technological domains? The goal of the study described in this paper is to investigate artifact interactions empirically and to test whether it has a retarding effect on improvement rates of technological domains. However, what are artifact interactions in a physical sense, and in what forms, do they manifest in artifacts?

In design of artifacts, Simon [11] introduced the notion of interactions in his essays on the complexity of artifacts. When a design of an artifact is changed from one state to another (with differences between the two states as defined by multiple attributes, say F1, F2, and F3) by taking some actions (say, A1, A2, and A3), in many cases, any specific action taken may affect more than one attribute, thus potentially manifesting as interactions of the attributes. In an artifact, attributes (F1, F2 and F3) can be interpreted as functional performance of a product, while actions (A1, A2, A3) can be interpreted as design parameters of components. This interpretation leads to the notion of interaction/conflicts as captured by the concept of coupling of functional requirements in Axiomatic Design [12], or dependencies between characteristics [13], which can occur when two or more functional requirements are influenced by a common design parameter, and are captured through tools such as Design Structure Matrix (DSM). Such coupling leads to the potential necessity of going back and forth negotiating before a new state is decided and thus introducing an interaction of the kind important in the models described above [8, 9, 10]. Theoretically, it would be ideal to have one design parameter controlling one functional requirement to achieve a fully decomposable (modular) design [12, 14]. Such fully decomposed design would not require any negotiation between components when changing from one state to another and thus would have minimal interactions.

Using an in-depth qualitative analysis of two technologies, VLSI (very-large-scale integration) systems and complex electro-mechanical-optical (CEMO) systems, Whitney [15, 16], however, has argued that the decomposability of a design of an artifact depends on the physics involved and/or additional design or resource constraints, such as permissible mass, and space. These aspects predispose some technological artifacts to be more decomposable than others. In other words, some technologies will inherently have more interactions than others will. Physically, these couplings or constraints manifest as component-to-component, and component-to-system interactions, or as a need to have multi-functional components. In such interactions, the component (or systems) involved affect other components and require negotiation in resolving constraints or complying with the physics involved. As illustration of the degree of interactions involved, and physical or design-related reasons behind them, Whitney noted that CEMO artifacts operate by processing significant amounts of power while VLSI artifacts operate by processing information, usually in electrical form, at very low levels of power. He showed that designers face fundamentally different challenges designing these two types of artifacts. The efficiency of CEMO artifacts depends on matching input and output impedances, which inherently creates interactions between the components, whereas VLSI designs decouple (that is, eliminate interactions between) the components by deliberately, hugely mismatching the impedances. Furthermore, high power levels create side effects at similar proportions, forcing CEMO designers to spend a large portion of their effort predicting and mitigating these side effects. These side effects manifest as additional interactions other than the functional ones designed between components (or systems). Whitney argues that CEMO systems additionally face several resource constraints on mass, size, and power consumption, forcing their designs to conserve these resources by utilizing multi-function components, further increasing inter- and intra-component interactions.

## Material and methods

### Approaches to study interactions in artifacts

Two potentially applicable approaches exist for studying artifact interactions. One method that is well recognized is the design structure matrix (DSM) [17], which when applied to products captures interactions between components in any artifact. The empirical method utilizes interviews with a broad variety of engineers who are knowledgeable about development of an artifact and are associated with effort on various components or systems that make up the artifact. Such interviews can capture geometrical, energy, material and information interactions and the DSM can be defined at different levels of abstraction of the product and the method has been well developed for some time now. If one could obtain reliable DSM data across a wide range of domains, this would be an effective way to study interactions. However, it is labor intensive to develop a DSM for a complex product such as a jet engine, an aircraft, and a MRI machine. Perhaps for this reason, the number of DSM publicly available in papers and at websites is limited. Further, it will be very hard, perhaps even impossible, to develop DSM of artifacts that were designed some time ago such as 30 years. Due to the scarcity of available data, (only one DSM is apparently available for a domain where the improvement rate is known), and the prohibitive cost of developing them, this approach was not pursued further to test the influence of interactions on improvement rate.

Another similar technique to represent interactions is networks with nodes and edges. Complex networks allow modeling and characterizing nonlinear behaviors underlying complex systems; for some novel and efficient complex network analysis theories, see [18–21]. For both DSM and network representation, information on artifact interactions first must be collected through techniques such as interviews or documents before it can be represented.

Another approach is to use documents: One set of documents potentially relevant for studying interactions are design manuals and engineering books related to specific domains, which could be analyzed using text mining techniques. Hommes and Whitney [22] have used documents describing product, and sub-systems level requirements to pursue case studies of system level interactions. Since very few documents describing interactions in artifacts belong to domains for which we have performance data, this was not a viable approach either.

Another set of promising documents are patents. Patents are particularly attractive; as a data source, they are generalizable, objective, and publically available. They provide meta-data, and qualitative data (drawings and text). The textual data describes state-of-art prior to the inventions, and associated problems that were solved. Second, the data is available for many generations of inventions, and easily accessible from USPTO or other websites such as Google.com. Additionally, the Classification Overlap Method (COM) [23, 24, 25], a recently developed tool based on UPC and IPC classification codes, enables identification of patents for each specific technological domain. Unlike in DSM of products in which interactions have already been identified, no interactions, however, are inherently defined in patents as patents are written for the protection of intellectual property, and patentability does not require interactions to be identified. Thus, it was necessary to develop a method for identifying and extracting interactions from patent documents.

### Procedure for text mining and analysis of patents

#### Procedure overview

The patent analysis using a text mining approach was conducted in two phases. In a pilot study, using patents from 5 domains—battery, wind power, solar PV, capacitors and computer tomography scanning (CT scan), feasibility for extracting data about artifact interactions from patent text was explored using Latent Semantic Analysis, Latent Dirichlet Analysis, and keyword-based techniques. Only the keyword-based technique was found to be useful in extracting data on interactions, and hence will be discussed further. In the extended study, the keyword-based technique developed in the pilot study was implemented in 27 domains.

Both the pilot and extended study consist of four broad steps: (1) preparation of textual data from patents (2) identification of interactions and associated keywords (3) keyword-based text mining (4) analysis and interpretation of variations in keywords across domains. In step 1, domain-specific patents were identified, electronically retrieved from the web, and cleaned to prepare text for analysis. In step 2, patents were read in detail to identify potential generic keywords associated with interactions. In step 3 the raw data on interactions was extracted using keyword-based text mining, which was then analyzed in step 4. Subsequent sections describe these four steps. Since step 2 is the most critical among these, we describe this step first.

#### Identification of artifact interactions and associated keywords (step 2)

Based on the qualitative work of Simon [11], Suh [12], Weber [13], and Whitney [15, 16] described above, we have classified interactions into three broad classes of interactions, which provide a useful framework for identifying text describing interaction and associated keywords. In The three classes of interactions are:

1. Between functional requirements: These interactions are consequences of the dependencies between multiple functions and design parameters [12]. For example, increasing the size of a mechanical component can increase its stiffness, a desirable quality. But, increasing size results in increase of mass, which can affect dynamics of the artifact adversely. When one function is improved, such interactions can lead other coupled functions to be adversely affected.
2. Between component and component, or between component and system: A good example of this type of interaction is the necessity to match impedance between sub-systems in order to maximize power transfer [15, 16].
3. Parasitic/side effects: These represent undesirable effects exhibited by the components and sub-systems, while they fulfill their main functions [15, 16]. Some examples of these are corrosion in battery electrodes, and heat dissipation in computers.

Using the above framework, as part of step 2 in the pilot study, two researchers, including the current lead author and an Intern working with the author for several months, carefully read a set of 60 patents from the 5 domains noted earlier (battery, capacitor, wind power, solar PV, and CT scan) to identify text describing technical issues that reflect interactions of the types discussed above. Three patents from each decade starting from the 1970’s until the present were selected to make a total of 12 patents in each of the 5 domains. It was observed that background or prior art sections, as expected, described problems with the state-of-art artifacts. It was also found that many patents, while summarizing the current invention, also discussed problems that were not previously discussed in the background or prior art section. In both of these sections, descriptions of problems consistent with the prior research [11–13, 15, 16] and with our classification scheme were accepted as interactions. The detailed description and claims sections focused on describing the current invention and novelties inventors wanted to claim as assignee’s intellectual property, and rarely included descriptions of interactions. Based on these findings in the pilot study, the decision was made to include text from the *title*, *abstract*, *background*, *and summary* sections, and not include the detailed description and claims sections to maximize signal to noise ratio.

The essential part of step 2 was to extract text samples that contained the description of the interactions. These text samples were examined for keywords that tended to appear as reliable signals of interactions but that were not domain specific. Two examples of such text (*italicized*) indicating interactions with associated keywords in ***bold*** are presented below in Table 1, set 1, and an extensive list is in the SI. Below each example text, our interpretation of the interaction using the framework and typology described above is presented. The first example text in the table may be interpreted as an interaction of functional requirements, specifically energy storage and preservation of electrical waveform. The second example clearly describes a side effect, where leakage of electrolyte leads to failure.

**Table 1:** Examples of text from patents describing interactions, and associated keywords.

	Patent text with keywords (in bold)	Interpretation of interactions
*Set 1*: *Keywords usage in patent text indicating an interaction*		
1	*Generally*, *conventional aluminum electrolytic capacitors have an energy storage value or capacitance which increases with applied voltage*. *This is probably due to penetration of the liquid electrolyte into the aluminum oxide surface coating on the anode*. *Sometimes*, *however*, *such penetration is* ***undesirable***, *as it can result in a change in the dielectric characteristics and hence in a distortion of the waveform in pulse applications*.	One function of this device—a capacitor—is to store energy, and another function is to preserve the waveform (applicable to some applications). The penetration described leads to increase in voltage, which improves the energy storage capability (first function); but at the same time, it deteriorates the second function, thus causing **interaction of the two functions**. The deterioration may also be viewed as an undesirable side effect.
2	*In such electrolytic capacitors there exists the risk that the liquid electrolyte will leak out*. *Accordingly*, *the capacitor must be hermetically sealed to* ***prevent*** *any leakage of the liquid electrolyte therefrom*, *since if the liquid were to come into contact with the other electronic components encapsulated in the device*, *it could damage them sufficiently to cause the device to fail to operate properly*.	The electrolyte, which has leaked, attacks neighboring components causing failure. Thus, this leakage and failure are **undesirable side effects**. The keyword that reflects this side effect is the word ‘**prevent**’.
*Set 2*: *Example of keyword usage* ***not*** *describing an interaction (irrelevant)*		
1	*A primary* ***problem*** *with most CT methods is that they are time consuming*. *Consequently*, *prior to this invention*, *CT technology has not been a feasible alternative to such* ***problem****s as screening luggage for concealed items*. *Screening luggage for concealed items is of vital importance*. *Such monitoring is necessary to avoid smuggling of drugs and to detect explosives planted in luggage by terrorists*. *Present techniques for screening luggage include manual inspection*. *Manual inspection is a time consuming and therefore expensive operation*.	This example text describes an unsatisfactory performance of the CT methods referenced by this patent, specifically slow speed of scanning. The usage of the keyword **‘problem’ does not indicate an interaction**. Second usage of the keyword is for an application, which may be seen as a design opportunity.

From reading of the 60 patents, a total of 30 keywords were found that potentially indicated interactions. These keywords were used to study 430 additional patents from the 5 domains for interactions. Using the raw data from text mining of these patents, three criteria were used to cull the keywords: (1) count of occurrence, (2) cross-domain usage of the keywords across the domains, and (3) relevancy of keywords in reflecting interaction. First, high occurrence of a keyword was necessary to get a statistically strong signal capable of showing variation across the domains. For example, the words ‘*problem*’ and ‘*prevent*’ were common keywords in description of technical issues. Second, since the goal of the study is to conduct a comparative study, it was also necessary to ensure that keywords were not domain specific but instead generally used. For this reason, the word ‘*corrosion*’ was not considered a good keyword, since it is too specific to particular domains, and may see no usage in many domains. The word ‘*prevent*’ or ‘*undesirable*’ was selected as a better choice in such instances, since it captures the notion of bad side effect that needs to be mitigated, but without being limited as to the detailed nature of the side effect. Third, it was also important to ensure that the keyword when it was used in text reflected interaction most of the time, that is the keyword has high sensitivity. This is assessed by relevancy of a keyword, which estimates how often a specific keyword reflects interaction when it is used in the text. This is defined as a ratio of count of keywords signaling interactions to the total count of the keyword usage in the patent set for a domain. Example patent text in Table 1, set 1 provides examples of relevant usage of the keywords. For comparison, the patent text in Table 1, set 2 provides an example of non-relevant usage of the keyword ‘problem’. The keyword ‘problem’ in this example is used to indicate inadequate performance and design opportunity, not interactions.

Data from the pilot study of 430 patents showed that majority of the 30 keywords words had low count, and hence were removed from the list. Additionally, using the cross-domain usage criteria, the remaining keywords were reduced to 8 keywords: ***parasitic***, ***problem***, ***prevent***, ***undesir***able, ***requirement***, ***fail***ure, ***disadvantag***e, and ***overcom***e. The root of each keyword searched is shown as bold and italicized text. The root is chosen so that the searched keyword is able to identify the maximum number of instances of the keyword, but without reducing the relevancy percentage. These 8 keywords had high count and are relatively general.

Relevancy percentages for the keywords were determined from reading of the 60 patents (described above) for identifying interactions and associated keywords. Mean relevancy percentages for these 8 keywords across the 5 domains are listed here inside the parentheses: *parasitic (97%)*, *problem (58%)*, *prevent (83%)*, *undesir*able (*94%*), *requirement (75%)*, *fail*ure (*72%*), *disadvantag*e (*81%*), and *overcom*e (*98%*). The relevancy percentage for each individual domain is presented in S3 Table. The keyword ‘overcome’ had indicated interactions in nearly all instances (on average 98% of the cases). In contrast, the keyword ‘problem’ has poor relevancy percentage; almost half the times (42%) it did not indicate interactions. This is because it is common among engineers to use the word ‘*problem*’ to describe design opportunities to improve main functions of a technology, or to take advantage of new applications. This convention is reflected in the patent text as exemplified by the example in Table 1, set 2. Because of the low relevancy percentages and its possibility to add much noise to the data, the keyword ‘*problem*’ was removed from the list.

**Fig 2:**
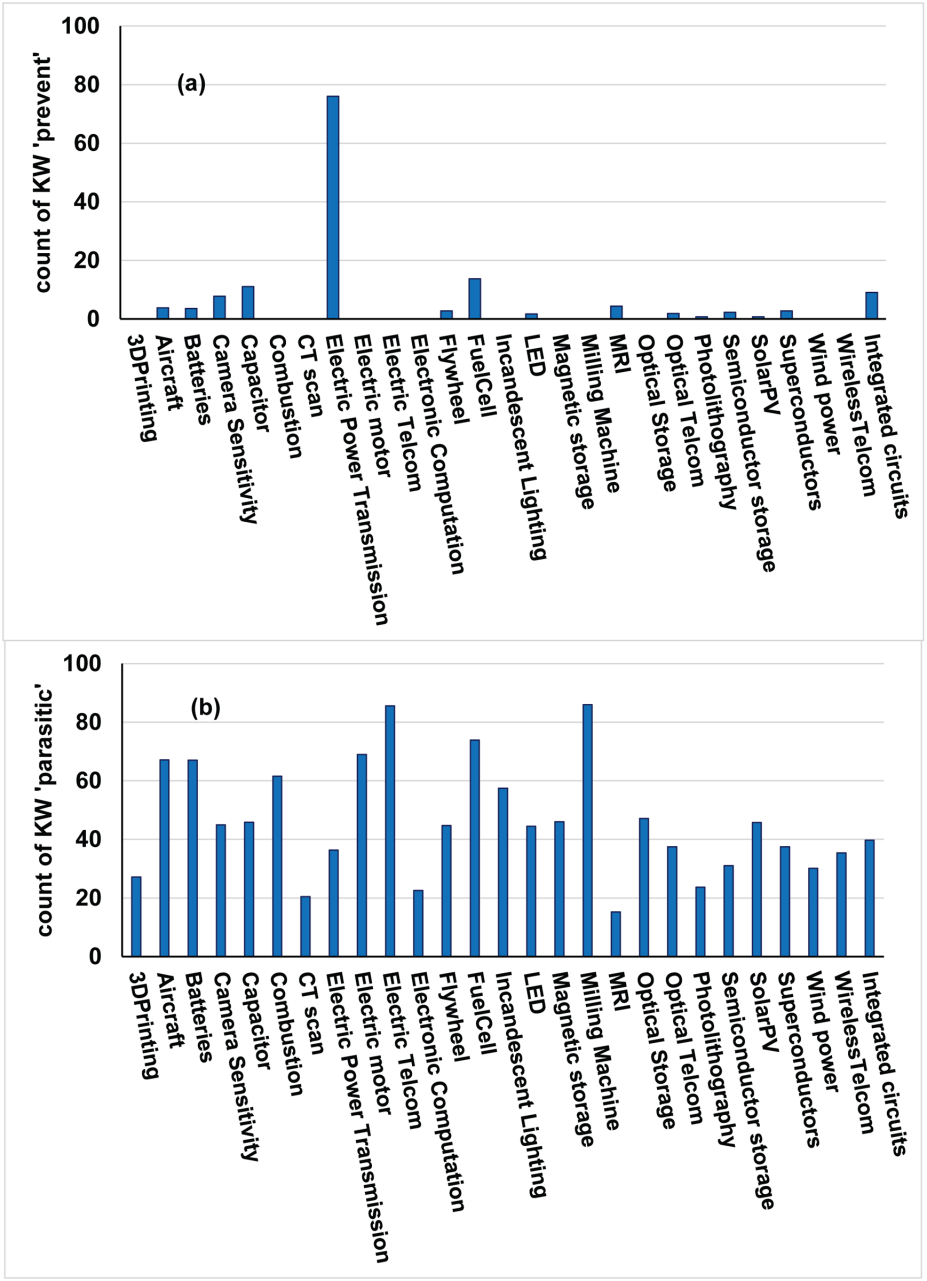
Cross-domain usage of keywords (KW) ‘parasitic’ (panel a) and ‘prevent’ (panel b) across 27 domains. The keyword ‘parasitic’ is not widely used; in fact, 12 domains do not use the ‘parasitic’ keyword at all. Compare this to wide cross-domain usage of keyword ‘prevent’. The count of KW presented is a normalized count of the respective keyword, with normalization carried out against 100 thousand total words.

The remaining 7 keywords were further vetted in the extended study with patents from 27 domains. The raw data from keywords showed that the keyword ‘parasitic’ did not have wide cross-domain usage. In fact, 12 domains did not use it even once, and only 5 domains—Camera Sensitivity, Capacitor, Electric Power Transmission, Fuel Cell, IC chips—used it often. Fig 2a shows the distribution of the “parasitic” keyword across all patents in 27 domains. To provide perspective, the distribution of the keyword “prevent” is presented in Fig 2b. It is clear the keyword “prevent” is used by all domains frequently. Due to a low cross-domain usage, the keyword ‘parasitic’ was also eliminated, leaving a final list of 6 keywords for cross-domain analysis.

#### Preparation of text from domain patents (step 1)

The outcome of this step was the raw text from the 100 most-cited patents for each of the domains being studied. (Please see S4 and S5 Tables for the list of patents used for each domain.) The following procedure was used to retrieve the text: (1) Patents in each specific technological domain were identified using the COM method [23, 24, 25]; out of which one hundred and fifty most-cited patents in each domain were chosen and read to eliminate any irrelevant patents from the set; then the 100 most-cited relevant patents were selected from the patent set. The patents had been identified as part of doctoral research [25] in which both authors of this paper participated in reading the patents for relevancy. (2) Four sections of the text—title, abstract, background, and summary—in each patent were downloaded from Google’s patent database. (3) To prepare the text for mining, extraneous text -such as stop words- was removed using Python scripts. Stop words are a set of commonly used words, such as *the*, *a*, *it*, and *in*. Although they are critical in natural language, they do not add any value to the data. Removing them makes it possible to focus on the important words, and to reduce computational cost as well as noise in the data. The completion of this step prepared the text for mining.

Out of the 2700 patents used for extended study (sub-step 2 above), 2400 patents were downloaded using a web-scraping tool from the Google patent database, which provides patents in searchable html files. Out of the remaining patents, we successfully downloaded 276 manually, thus resulting in 97 to 100 patents in each domain. Almost all of these manually downloaded patents either lacked proper titles describing the sections, or background and summary were merged with detailed descriptions. For these cases, the background information and summary had to be identified by reading the patents and then manually extracted.

#### Text mining for keywords (step 3) and analysis (step 4)

The prepared patent text for the 27 domains was mined using Python scripts for determining the count of the keywords as well as the total number of words in the patent set in each domain. The total number of words in the patent set was used for normalizing the count of keywords in each domain, and the normalized keyword count is expressed per 100 thousand words in the patent set.

For analysis, we assumed a linear relationship between normalized count of keywords and artifact interactions described by the models [8, 9, 10]. We then tested dependence of improvement rates on artifact interactions as predicted by the models by conducting a correlation analysis of improvement rates and normalized keyword count from the patent text for the 27 domains.

## Results and analysis

### Total count of words across domains

The total count of words (all text including keywords) in patent text varies widely between the individual patents, with the ratio of total word count between the patent with the highest word count to the patent with the lowest count was more than 100. However, when the total word count of domain-specific patent sets is compared, the distribution of total word count is much narrower, shown in S1 Fig. The domain-level total word count ranges from roughly 191,000 down to 95,000, a ratio of slightly over two. The five domains with the highest total word count in descending order are genome sequencing, 3D printing, optical memory, CT scan, and wireless telecommunications. The domains with lowest count in ascending order are electric motor, electrical telecommunications, milling machine, optical telecom, and flywheel. Since domains with more text can potentially have higher occurrence of keywords, the variation in total word count between domain patent sets indicates that it is necessary to normalize keyword count with respect to total word count. Reading of genome sequencing patents shows they have much higher occurrence of chemical formulas than any other domain. Since keywords representing interactions are not found in these formulas, the normalized count was highly distorted for this domain so the domain is eliminated in all analysis reported here.

### Normalized count of keywords (KW)

**Fig 3:**
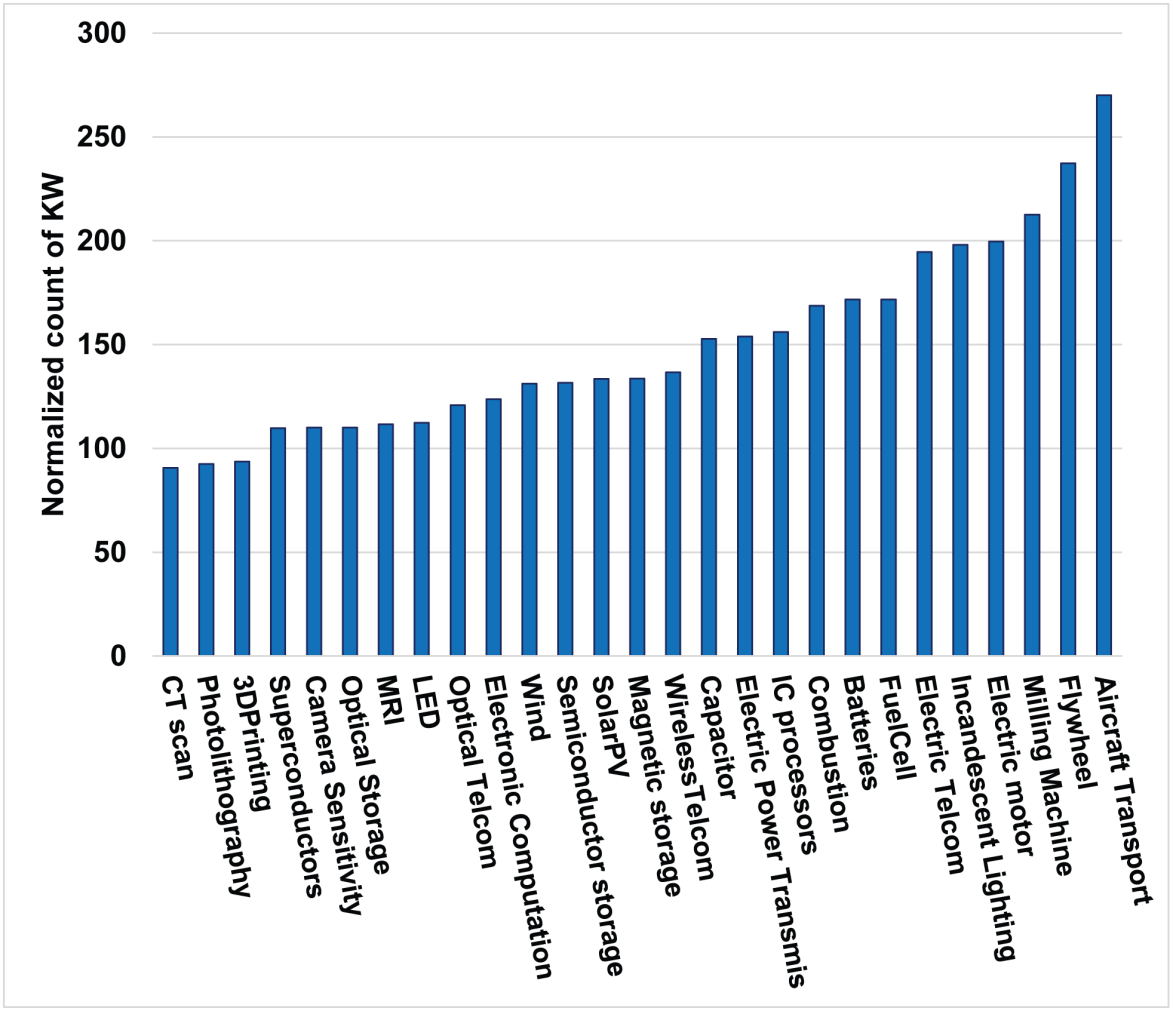
Comparison of count of normalized 6-keywords (KW) for 27 domains. The count of KW is the normalized total count of 6 keywords identified to indicate interactions in the text (abstract, title, background, and summary of invention) from the 100 most-cited patents in each domain, where normalization is carried out with respect to total number of all words in the text, and expressed per 100 thousand words.

The count of the 6 keywords is summed, normalized with respect to total word count in the respective patent set, and finally expressed in terms of keywords per 100,000 words. Fig 3 shows the distribution of the normalized count of 6 keywords across the 27 domains. The five domains with the highest normalized 6-keyword count in descending order are Aircraft, Electric power transmission, Flywheel, Electric telecommunication, and Milling machine. The domains with the lowest count in ascending order are CT scan, Superconductors, MRI, 3D Printing-SLA.

### Correlation analysis of improvement rates and reciprocal of normalized count of keywords

According to the model [8, 9, 10], the improvement rates should be inversely proportional to the interaction parameter *d*_*J*_. The normalized keyword count is assumed to provide a relative estimate of artifact interactions encountered in generating inventions in the various domains: we assume a linear relationship between the normalized keyword count (KW_J_) and the degree of interactions characteristic of a domain (d_J_). With this assumption, the prediction based upon Eq (2) then becomes

$$K_{J}=\frac{d\;lnQ_{J}}{dt}\;\propto \;\frac{1}{d_{J}}\;\propto \;\frac{1}{KW_{J}}$$ (3)

Relationship 3 is expressed as a hypothesis as follows:

Hypothesis: *The performance improvement rate of technological domains are positively correlated with the inverse of the normalized count of interaction keywords in a set of patents belonging to each of the domains*.

**Fig 4:**
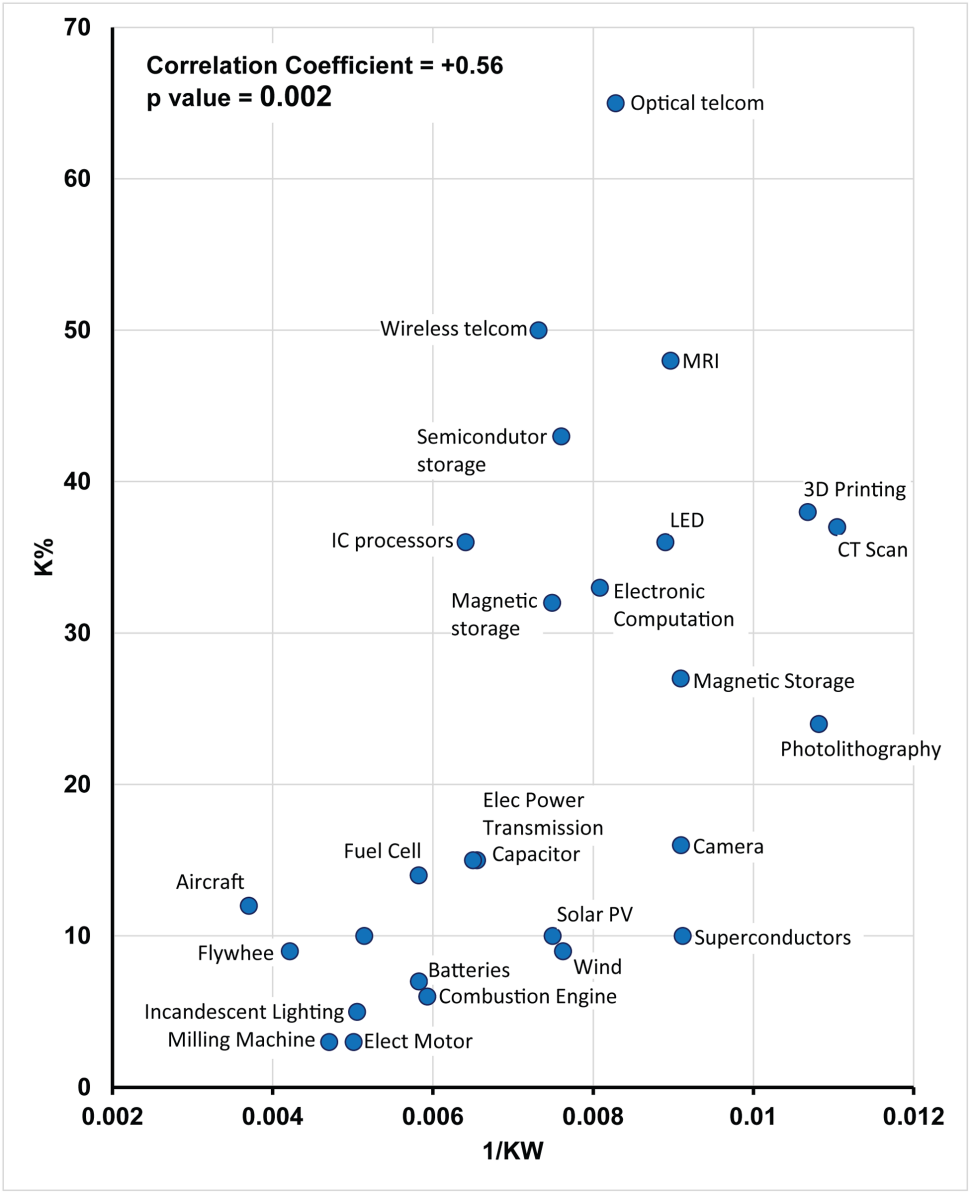
Scatter plot of K_J_ (annual improvement rates) and 1/KW (reciprocal of normalized count of keywords for 27 domains. Improvement rates are positively correlated with Pearson correlation coefficient = +0.56 with p-value = 0.002, and Spearman Rank Order correlation = +0.632 with p-value = 0.001. Note that capacitor and electric power transmission data points overlap, and hence appear as one point. The dashed line shows a linear trend line between K_J_ and 1/KW.

The hypothesis was tested empirically using the 6-keyword results. The improvement rate in a domain is plotted as a function of the inverse of normalized count of the 6-keywords in the same domain in Fig 4. Although there is significant scatter in the data, a clear upward trend can be visually observed, implying that higher improvement rates (K_J_) positively correlate with higher values of the inverse of normalized count of the 6-keywords (KW_J_).

The correlation coefficients using two widely used methods, Pearson (parametric method) and Spearman Rank Order (non-parametric method), have been calculated. Assuming a normality of the population of K values and of 1/normalized count of KW, the Pearson correlation coefficient calculated using EXCEL2013 is +0.56 with a p-value of 0.002. The Spearman’s Rank Order coefficient is 0.632 with p-value slightly less than 0.001. Note that the Spearman’s correlational method does not assume any distribution. Both p-values are much smaller than 0.05, a threshold value employed by many researchers; the value of 0.002 and 0.001 indicate a very strong likelihood that the correlation is not due to random effects. This result supports the theoretical prediction that domains associated with higher degree of interactions improve at a slower pace. How reliable is this correlation? We further examine the reliability of this finding using a robustness study using Pearson’s correlation coefficients.

### Robustness test

The robustness study was conducted by creating 20 groups of 14 domains (about half of the total number of domains in the study), where each group was generated by randomly selecting a combination of 14 domains from the 27 domains. For each group of 14 domains, the Pearson’s correlation coefficient between K and 1/KW was calculated.

**Fig 5:**
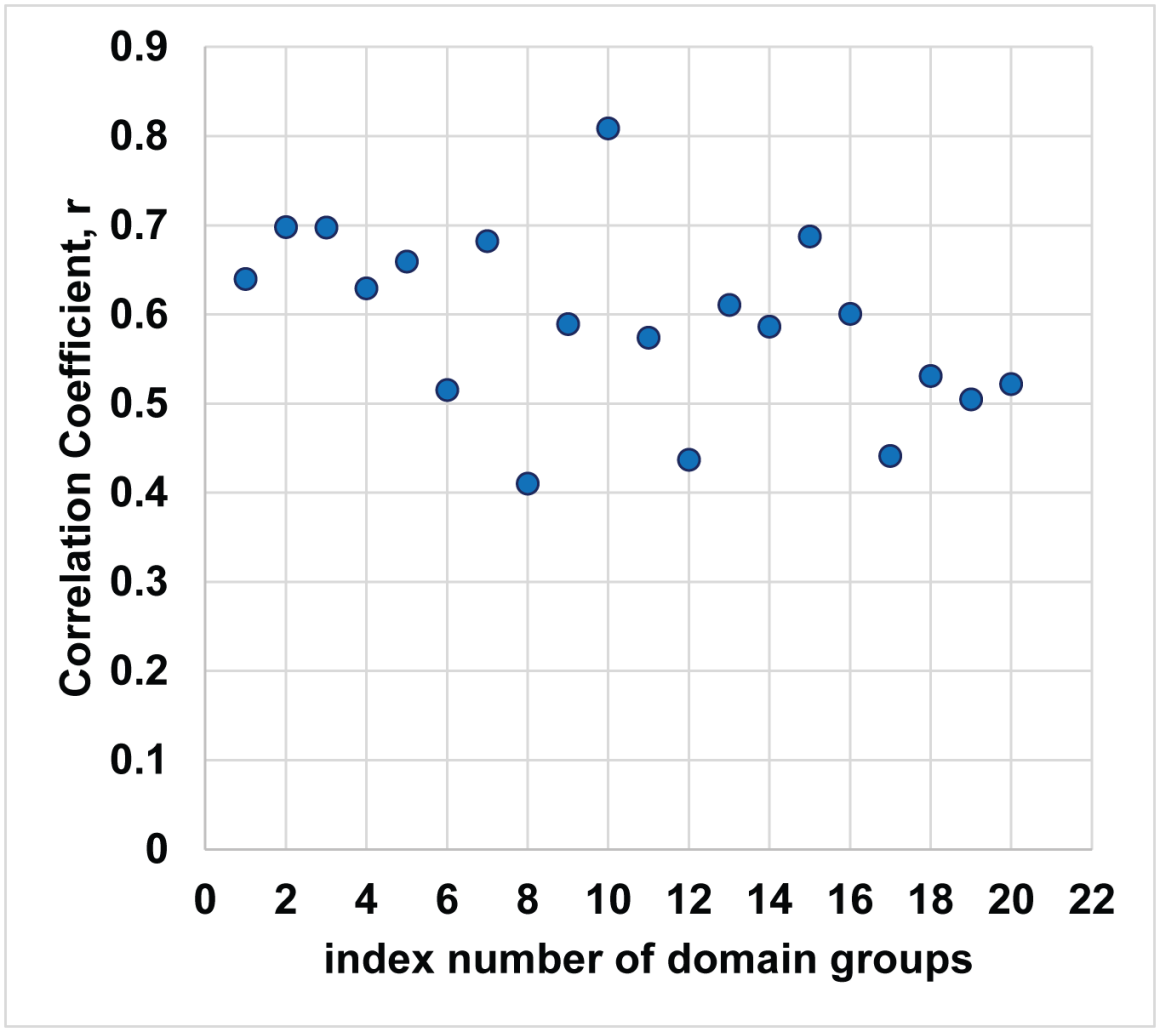
Scatter plot of Pearson correlation coefficients for K_J_ (improvement rates) and 1/KW (reciprocal of normalized count of keywords) for 20 groups. The average correlation coefficient (r) is +0.59 with standard deviation of 0.10. The coefficient value for 27 domain as a whole is 0.56. Each group consists of a combination of 14 randomly selected domains from the 27 domains.

The results of those 14 groups are presented in Fig 5 with the index number of each group plotted along the X-axis, and Pearson’s correlation coefficient along the Y-axis. It is clear from the figure that the correlation values are all positive and range relatively narrowly from +0.41 to +0.81. The average correlation coefficient value is +0.59. These results show that the correlation value is relatively stable, and further supports the conclusion that the correlation coefficient that was obtained in the initial study (+0.56) was not due to random effects or errors associated with particular domains.

### Discussion

The goal of this study was to investigate artifact interactions empirically, and to test the theoretical prediction in Eq (2) that interactions associated with technological domains retard their performance improvement rates. The qualitative descriptions of interactions described in the previous literature [11–13, 15, 16], specifically, component-to-component (or component to system), conflict between functional requirements, and side effects, were utilized as the foundation for investigating artifact interactions in textual content in patent sets for different domains. The normalized count of selected keywords associated with patent text reflecting artifact interaction was used to estimate the degree of artifact interactions in the technological domains. The correlation study found that performance improvement rates are positively related to the inverse of the normalized keyword counts. In other words, the study empirically demonstrates that artifacts interaction has a retarding effect on improvement rates.

The first methodological finding of the empirical study is that patents can be a useful resource for studying artifact interactions, and to our knowledge, this is the first time patents have been used to study interactions. This finding potentially opens up the possibility of using patents as a new generally available data source for studying artifact interactions and other similar attributes in artifacts, including those designed many decades in the past.

The seconding methodological finding is that the selected six keywords–*prevent*, *undesir*able, *requir*ement, *fail*ure, *disadvantag*e and *overcom*e—signal different types of interactions: component-to-component, component-to-system, side effects, and coupling of functional requirements. The keywords have been selected based on three criteria: (1) that they have at least a moderate level of occurrence (that the signal is strong enough); (2) that they are widely used across domains hence requiring them to be non-domain specific; (3) that they signal interaction most of the time in the text in which they are used. In order to satisfy criterion 2, keywords are chosen such that they describe interactions at a higher abstraction level. For an example, usage of the keyword ‘prevent’ implies prevention of some problem, but without specifying what the problem is. The further specification (such as a word ‘corrosion’) would potentially make it domain specific and not useful in the kind of cross-domain test performed here. Importantly, criterion 3 requires that the keyword is relevant, that is, the text marked by the selected keywords needs to signal interactions most of the time (true positives). The difficulty arises when the keyword used may have two (or more) meanings, such that the text in which it is used signals interaction in one case, but not in the other. The keyword ‘problem’ discussed earlier (see Material and methods) represents such a word. This keyword was screened out because it was associated with text reflecting design opportunities 44% of the time, although the other 56% of the time, it represented interactions. The work reported here demonstrates that such keywords can be found and the six utilized here are demonstrated to be applicable to a fairly diverse range of 27 technological domains.

The analysis showed that improvement rates and the inverse of normalized keyword counts are positively correlated with the Pearson coefficient (r) equal to +0.56 with p-value equal to 0.002, and Spearman rank order coefficient of 0.632 with p-value 0.001. The correlation is medium, and does not explain all the variation in the rates. This can be understood in the context of Eq (2). According to the model, another factor contributing towards variation of improvement rates is the scaling of design variables. Assuming that the influence of scaling (A_J_) on improvement rates across the 27 domains is as indicated by the Eq (2), the equation implies that improvement rates and inverse of keywords by itself can be expected to have medium correlation.

Although this work is the first of its kind in using patents to study domain interactions, the approach has some limitations. First, it can be observed that the noise is quite significant. Although much of this could be due to the other missing theoretical variable, scaling or to inaccuracy in measuring K_J_, another very likely source is due to limited resolution of the keywords as an estimate of interactions. An additional possible source of noise might be due to the limited number of patents (100 most-cited patents) being used for the study (due to limited resolution of Classification Overlap Method (COM) in its current state (Material and methods)). This issue should be more significant for domains with low normalized count of keywords, which is consistent with the higher spread of data points at lower count of keywords in the graphs (see right side Fig 4). An open issue is whether the keywords we have identified would work to reliably estimate interactions in domains that we have not examined. Although the keywords were carefully selected to be general, we did find that the estimate was distorted in a domain with extensive use of chemical symbols. In this study, the only problematic domain was genome sequencing but other domains could have this specific issue or other text “anomalies” that badly interfere with obtaining a meaningful estimate. Further research on more domains including reading patents would increase our understanding of the generalizability of the method.

Overall, the correlation analysis from this empirical study strongly supports the theoretical prediction that the domain interaction parameter is a factor that can lead to variation in improvement rates, where a higher interaction parameter leads to lower improvement rates. Further, it also supports the relational form the model predicts.

## Supporting information

S1 Table Search terms used for identifying the sections in the Google patent database. The search terms are presented in column 2 between quotes. Asterisk (*) represents a wild card character used in Python code for searching multiple characters in text strings.
(DOCX)

S2 Table Summary of data from empirical study of interactions in 28 domains. Columns 1 and 2 identify the domains; column 3 lists number of patents used in domains; columns 4–9 list count of 6 keywords (*prevent*, *undesirable*, *requirement*, *fail*, *disadvantage*, *overcome*) followed by cumulative count of 6 keyword for each domain; columns 11 lists total words in each domain, then followed by normalized count of 6 keywords, and performance improvement rate (K_J_).
(DOCX)

S3 Table Relevancy of keywords in 5 domains, and their average across 5 domains. The relevancy is the ratio of frequency of the keyword actually signaling interactions to frequency of keyword. The character ‘/’ in the table indicates that the specific keyword was not found in the text studied.
(DOCX)

S4 Table List of technological domains and their associated patent identification numbers. S4 Table lists technological domains and their associated patents which were studied. For each technological domain, column 3 provides the range of patent identification numbers associated with the respective domain. The S5 Table then presents an identification number and the associated patent along with its Google URL.
(XLSX)

S5 Table List of patent identification numbers from S4 Table with corresponding patents. (XLSX)

S1 Fig Variation of count of all words per domain. The count of words includes count keywords and all other words in the text (abstract, title, background, and summary of invention) from approximately 100 most-cited patents in each domain. This, however, does not include the count of stop words (e.g., articles etc.) removed from the text. S3 Table. Relevancy of keywords in 5 domains, and their average across 5 domains; character ‘/’ indicates no keywords were found in the text studied.
(DOCX)

S1 Text Additional examples of patent text using the six keywords. (DOCX)
