# Supplementary material for: Artifact interactions retard technological improvement: An empirical study
Source: PLoS One. 2017 Aug 4;12(8):e0179596. doi: 10.1371/journal.pone.0179596 (PMC5544181; doi:10.1371/journal.pone.0179596)
Supplement: S1 Table — The search terms are presented in column 2 between quotes. Asterisk (*) represents a wild card character used in Python code for searching multiple characters in text strings. (DOCX) [file pone.0179596.s001.docx]

**S1 Table. Search terms used for identifying the sections in the Google patent database.**

|  | **Section name** | **Terms used for searching section headers** |
| --- | --- | --- |
| 1 | Title | ‘Patent-title’  ‘Invention-title’ |
| 2 | Abstract | ‘Abstract’ |
| 3 | Background  (for exact match in heading) | 'description of the prior art',  'background of the invention',  'background',  'background information',  'prior art',  'introduction to the invention' |
|  | Background  (for partial match in heading) | '.*background.*',  '.*prior art.*',  '.*related technology.*',  '.*related art.*' |
|  | Background  (for partial match in paragraph) | '.*background.*',  '.*prior art.*',  '.*related art.*' |
| 4 | Summary  (for exact match in heading) | 'summary of the invention',  'statement of the invention',  'general description of the invention',  'brief description of the invention',  'short description of the invention',  'brief description of the present invention' |
|  | Summary  (for partial match in heading) | '.*summary.*' |
|  | (for partial match in paragraph) | '.*summary.*',  '.*statement of the invention.*',  '.*general description of the invention.*',  '.*brief description of the invention.*' |
