# Supplementary material for: Artifact interactions retard technological improvement: An empirical study
Source: PLoS One. 2017 Aug 4;12(8):e0179596. doi: 10.1371/journal.pone.0179596 (PMC5544181; doi:10.1371/journal.pone.0179596)
Supplement: S2 Table — Columns 1 and 2 identify the domains; column 3 lists number of patents used in domains; columns 4–9 list count of 6 keywords (prevent, undesirable, requirement, fail, disadvantage, overcome) followed by cumulative count of 6 keyword for each domain; columns 11 lists total words in each domain, then followed by normalized count of 6 keywords, and performance improvement rate (KJ). (DOCX) [file pone.0179596.s002.docx]

**Table S2: Summary of data from empirical study of interactions in 28 domains.**

|  | | | **Count of individual keywords** | | | | | |  | | | |
| --- | --- | --- | --- | --- | --- | --- | --- | --- | --- | --- | --- | --- |
| **Domain #** | **Domain name** | **Number of patents** | **Prevent** | **Undesirable** | **Requirement** | **Fail** | **Disadvantage** | **Overcome** | **6KW, total** | **words, total** | **(6KW/ Words) * 1e5** | **K_J_%** |
| Domain_1 | 3DPrinting | 100 | 47 | 14 | 31 | 11 | 45 | 14 | 162 | 172952 | 94 | 38 |
| Domain_2 | Aircraft Transport | 100 | 88 | 14 | 81 | 99 | 48 | 24 | 354 | 131060 | 270 | 12 |
| Domain_3 | Batteries | 100 | 75 | 8 | 48 | 28 | 18 | 15 | 192 | 111825 | 172 | 7 |
| Domain_4 | Camera Sensitivity | 99 | 58 | 3 | 34 | 3 | 25 | 19 | 142 | 129106 | 110 | 16 |
| Domain_5 | Capacitor | 100 | 54 | 23 | 30 | 25 | 32 | 16 | 180 | 117888 | 153 | 15 |
| Domain_6 | Combustion | 99 | 69 | 12 | 23 | 41 | 22 | 22 | 189 | 112038 | 169 | 6 |
| Domain_7 | CT scan | 100 | 31 | 16 | 21 | 14 | 32 | 23 | 137 | 151289 | 91 | 37 |
| Domain_8 | Electric Power Transmission | 100 | 42 | 15 | 48 | 28 | 32 | 13 | 178 | 115704 | 154 | 15 |
| Domain_9 | Electric motor | 99 | 66 | 20 | 29 | 13 | 42 | 21 | 191 | 95661 | 200 | 3 |
| Domain_10 | Electric Telcom | 100 | 88 | 9 | 36 | 25 | 26 | 16 | 200 | 102817 | 195 | 10 |
| Domain_11 | Electronic Computation | 99 | 33 | 0 | 58 | 62 | 19 | 9 | 181 | 146260 | 124 | 33 |
| Domain_12 | Flywheel | 100 | 48 | 11 | 39 | 80 | 54 | 23 | 255 | 107438 | 237 | 9 |
| Domain_13 | FuelCell | 99 | 108 | 14 | 73 | 11 | 28 | 17 | 251 | 146123 | 172 | 14 |
| Domain_14 | Genome sequencing | 99 | 42 | 2 | 16 | 7 | 21 | 13 | 101 | 191484 | 53 | 29 |
| Domain_15 | Incandescent Lighting | 100 | 63 | 15 | 21 | 62 | 42 | 14 | 217 | 109610 | 198 | 5 |
| Domain_16 | LED | 100 | 53 | 7 | 12 | 16 | 29 | 17 | 134 | 119257 | 112 | 36 |
| Domain_17 | Magnetic storage | 99 | 64 | 7 | 43 | 17 | 26 | 29 | 186 | 139223 | 134 | 32 |
| Domain_18 | Milling Machine | 97 | 89 | 16 | 28 | 22 | 37 | 28 | 220 | 103482 | 213 | 3 |
| Domain_19 | MRI | 98 | 21 | 14 | 24 | 17 | 58 | 20 | 154 | 138033 | 112 | 48 |
| Domain_20 | Optical Storage | 99 | 72 | 3 | 19 | 34 | 31 | 9 | 168 | 152731 | 110 | 27 |
| Domain_21 | Optical Telcom | 99 | 40 | 7 | 31 | 6 | 23 | 22 | 129 | 106801 | 121 | 65 |
| Domain_22 | Photolithography | 98 | 33 | 27 | 31 | 11 | 13 | 14 | 129 | 139494 | 92 | 24 |
| Domain_23 | Semiconductor storage | 97 | 41 | 7 | 28 | 30 | 47 | 21 | 174 | 132235 | 132 | 43 |
| Domain_24 | SolarPV | 98 | 59 | 11 | 42 | 25 | 22 | 13 | 172 | 128842 | 133 | 10 |
| Domain_25 | Superconductors | 100 | 41 | 14 | 20 | 11 | 15 | 19 | 120 | 109385 | 110 | 10 |
| Domain_26 | Wind | 99 | 39 | 8 | 31 | 29 | 34 | 29 | 170 | 129593 | 131 | 9 |
| Domain_27 | WirelessTelcom | 99 | 52 | 8 | 60 | 19 | 33 | 29 | 201 | 147087 | 137 | 50 |
| Domain_28 | Integrated Circuits (IC) | 99 | 44 | 11 | 54 | 13 | 37 | 14 | 173 | 110844 | 156 | 36 |
