# Supplementary material for: Artifact interactions retard technological improvement: An empirical study
Source: PLoS One. 2017 Aug 4;12(8):e0179596. doi: 10.1371/journal.pone.0179596 (PMC5544181; doi:10.1371/journal.pone.0179596)
Supplement: S3 Table — The relevancy is the ratio of frequency of the keyword actually signaling interactions to frequency of keyword. The character ‘/’ in the table indicates that the specific keyword was not found in the text studied. (DOCX) [file pone.0179596.s003.docx]

**S3 Table. Relevancy of keywords in 5 domains, and their average across 5 domains**.

| **Keywords** | **Arithmetic mean** | **Batteries** | **Wind** | **PV** | **Capacitors** | **CT scan** |
| --- | --- | --- | --- | --- | --- | --- |
| ***Parasitic'*** | 0.97 | 0.99 | / | 0.99 | 0.94 | / |
| ***problem*** | 0.58 | 0.68 | 0.61 | 0.55 | 0.5 | 0.55 |
| ***prevent*** | 0.83 | 0.93 | 0.85 | 0.76 | 0.85 | 0.75 |
| ***undesir***able | 0.94 | 0.88 | 0.95 | 0.99 | 0.99 | 0.875 |
| ***requirement*** | 0.75 | 0.85 | 0.69 | 0.79 | 0.81 | 0.63 |
| ***fail***ure | 0.72 | 0.92 | 0.68 | 0.78 | 0.74 | 0.5 |
| ***disadvantag***e | 0.81 | 0.93 | 0.8 | 0.79 | 0.8 | 0.71 |
| ***overcom***e | 0.98 | 0.99 | 0.96 | 0.99 | 0.95 | 0.99 |
