# Supplementary material for: Artifact interactions retard technological improvement: An empirical study
Source: PLoS One. 2017 Aug 4;12(8):e0179596. doi: 10.1371/journal.pone.0179596 (PMC5544181; doi:10.1371/journal.pone.0179596)
Supplement: S1 Fig — The count of words includes count keywords and all other words in the text (abstract, title, background, and summary of invention) from approximately 100 most-cited patents in each domain. This, however, does not include the count of stop words (e.g., articles etc.) removed from the text. S3 Table. Relevancy of keywords in 5 domains, and their average across 5 domains; character ‘/’ indicates no keywords were found in the text studied. (DOCX) [file pone.0179596.s006.docx]

**S1 Fig. Variation of count of all words per domain.** The count of words includes count keywords and all other words in the text (abstract, title, background, and summary of invention) from approximately 100 most-cited patents in each domain. This, however, does not include the count of stop words (e.g., articles etc.) removed from the text. S3 Table. Relevancy of keywords in 5 domains, and their average across 5 domains; character ‘/’ indicates no keywords were found in the text studied.
